# Practical Departments

**Published:** 1897-04-03

**Authors:** 


### PRACTICAL DEPARTMENTS.

*Small Cottage Hospitals.—A correspondent writes: "It is proposed to start a Cottage Hospital in a district which has a population of about 6,000. It is proposed, however, to start with only four beds, and it has been estimated that the maintenance will amount to only £120, including everything except rent—that is, £30 per bed. Will you kindly express an independent opinion on the matter?"*

It is quite clear to us, having regard to the contingent expenses and unforeseen matters which are sure to arise in a new institution, that such a hospital ought to have an income of £200, or at least £180. Of course, local circumstances and the extent to which support may be given in kind by the inhabitants of a district materially affect the actual cost of maintenance, but in all the circumstances we are of opinion that £200 is a reasonable estimate if the nursing arrangements are to be adequate and the staff be properly paid, as it is necessary it should be. An income of £180 might be sufficient, but it is best to over-estimate rather than to under-estimate the preliminary expense of a new institution so as to avoid any disaster.

